# Physical Mistreatment in Persons with Alzheimer's Disease

**DOI:** 10.1155/2013/920324

**Published:** 2013-03-17

**Authors:** Carla VandeWeerd, Gregory J. Paveza, Margaret Walsh, Jaime Corvin

**Affiliations:** ^1^The Harrell Center for The Study of Family Violence, Department of Community and Family Health, College of Public Health, University of South Florida, 13301 Bruce B. Downs Boulevard, MDC 56, Tampa, FL 33612, USA; ^2^School of Health and Human Services, Southern Connecticut State University, 501 Crescent Street, Lang House, New Haven, CT 06515, USA; ^3^Department of Community and Family Health, College of Public Health, University of South Florida, 13301 Bruce B. Downs Boulevard, MDC 56, Tampa, FL 33612, USA; ^4^Department of Global Health, College of Public Health, University of South Florida, 13301 Bruce B. Downs Boulevard, MDC 56, Tampa, FL 33612, USA

## Abstract

Physical mistreatment has been estimated to affect 2 million older persons each year and dramatically affects health outcomes. While researchers have attempted to examine risk factors for specific forms of abuse, many have been able to focus on only victim or perpetrator characteristics, or a limited number of psychosocial variables at any one time. Additionally, data on risk factors for subgroups such as persons with Alzheimer's disease who may have heightened and/or unique risk profiles has also been limited. This paper examines risk for physical violence in caregiver/patient dyads who participated in the Aggression and Violence in Community-Based Alzheimer's Families Grant. Data were collected via in-person interview and mailed survey and included demographics as well as measures of violence, physical and emotional health, and health behaviors. Logistic regression analysis indicated that caregivers providing care to elders with high levels of functional impairment or dementia symptoms, or who had alcohol problems, were more likely to use violence as a conflict resolution strategy, as were caregivers who were providing care to elders who used violence against them. By contrast, caregivers with high self-esteem were less likely to use violence as a conflict resolution strategy. Significant interaction effects were also noted.

## 1. Introduction

While child abuse has been recognized and studied in the literature in depth, in the past 30 years researchers have begun to recognize the vulnerability of older adults to this issue and to increase the scope of abuse research to include the study of mistreatment in older persons. Mistreatment of older adults has been associated with age and gender of victim [1–6] with the oldest old and women found to be at significant risk. It has been linked to domestic violence theories with spouses often found to be the most likely perpetrators [7–9]. Heightened risk has been linked to increased stress, with caregivers financial [10–12] or emotional dependence [9, 13–15], marital discord, and financial difficulties [16] increasing the likelihood that abuse will happen.

Caregiver and care receiver isolation have also been associated with elder mistreatment [15, 17–20], as have inadequate exchange issues such as violence by care recipients [21–27] and poor caregiver/care receiver relationships [22, 26, 28–30]. Caregiver psychopathology such as substance abuse, depression [9, 31–36], and caregiver emotional problems [29, 31, 36, 37] have also been linked [36, 38, 39]. Care receiver cognitive impairment has recently received attention as a risk factor for elder mistreatment [23, 26, 40–46].

Physical abuse involves acts of violence that may result in pain, injury, impairment, or disease [22, 47]. Physical mistreatment dramatically affects the lives of older adults and it has been estimated to affect as many as 2 million [48]. Further it is estimated that 2–10% of older adults may be victims of abuse, with only 1 out of every 14 incidents reported [49]. While researchers have attempted to look for risk factors of specific forms of mistreatment such as physical abuse [11, 12, 21, 50–52] many have been able to utilize only victim or perpetrator characteristics, or to focus on a limited number of psychosocial indicators at any one time. The ability to study subgroups of older adults who might be at increased risk for elder mistreatment as a result of special needs has also been limited. While researchers have begun examining subgroups, such as persons with dementia who may have increased risk for physical mistreatment as result of the etiology of their disease [42, 53, 54], the relationship between elder mistreatment and persons with Alzheimer's Disease (AD) has been largely understudied as a result of difficulties in finding and accessing these populations on a large scale. As a result, the ability to study multiple caregiver and care recipient risk factors in these populations simultaneously has often been curtailed. 

What is known is that elder mistreatment results in family distress, impaired life functioning [55, 56], and cognitive difficulties [57, 58]. It results in emotional difficulties such as feelings of inadequacy, and self-contempt [37, 59, 60], decreased self-esteem [57, 59–63] and depression [56, 64, 65]. It has also been linked to health problems such as increased mortality [66–68] and immunological dysfunction [56, 57, 63, 65, 69, 70]. As such, it is imperative that we begin to study it not only in older adults in general, but also in specific subgroups who may have unique characteristics that result in a differing risk profile.

**Figure 1:**
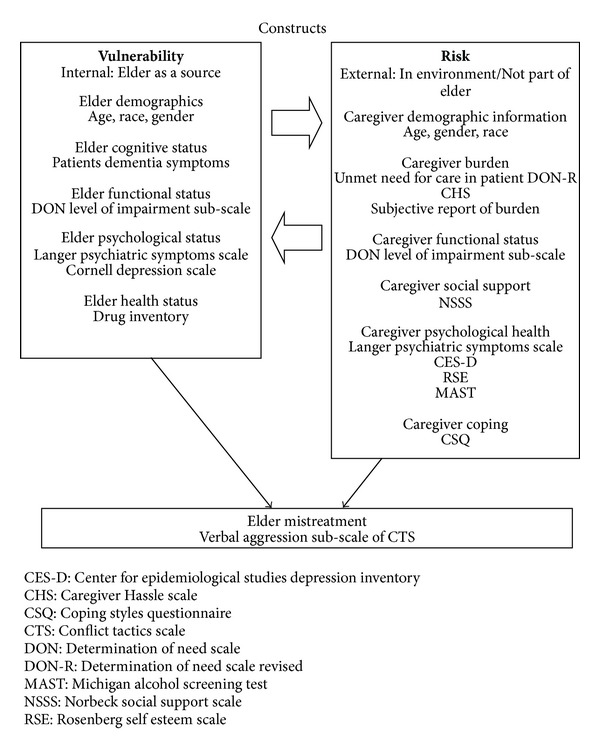
A Risk Vulnerability Model of Elder Mistreatment.

This paper will look at risk factors for physical mistreatment in a sample of community dwelling older adults with Alzheimer's Disease in the state of Florida using the risk and vulnerability model of Elder Mistreatment (see Figure 1). The Risk and Vulnerability model proposed by Rose and Killian (1983) [71] was first applied to elder mistreatment in 1994 by Frost and Willette, and then to neglect by Fulmer and Paveza [72]. While many studies conducted to date focus only on victim or perpetrator characteristics, what is unique about this model is its ability to see both as contributing to the likelihood that abuse will happen. Risk under the model refers to hazards or stressors in the environment external to the elder that contribute to likelihood of mistreatment, while vulnerability refers to characteristics within an elder that may influence the likelihood that abuse happens. For a person with Alzheimer's disease, vulnerability may stem from factors such as decreased cognitive status, increased levels of functional impairment, as well as behavioral difficulties, which stem from the etiology of their disease and result in the need for a primary caregiver, and add “risk” in the form of caregiver burden, poor functional and psychological health, and difficulties in coping. This model postulates that abuse may stem from several areas in which AD persons might be susceptible and offers a framework to test several risk factors for mistreatment simultaneously (Figure 1).

## 2. Methods

### 2.1. The Data Set

This study represents a secondary analysis of data collected as part of the Aggression and Violence in Community Based Alzheimer's families grant (AV_CAD) described in detail in VandeWeerd and Paveza (2006) [73]. Potential subjects were enrolled though solicitation via membership in one of 3 local chapters of the Alzheimer's Association located in Tampa, Orlando, and Miami, or through their participation with the 5 state funded memory disorder clinics (Tampa, Central Florida, North Broward, Miami, and Miami Beach) over a 3 step procedure (see Box 1).In step one, all older adults diagnosed with AD who were receiving treatment from one of the memory disorder clinics (*n* = 1,781), or who were members of Alzheimer's Associations during the studies enrollment period (*n* = 5,648) were mailed an introductory pamphlet explaining the study. Pamphlet packages included a response card indicating a person's interest in the study and their consent to have their information released from their enrollment site to the study team. In step two, all interested persons were contacted by the USF study team who explained the study in detail and verified whether the elder and caregiver met the inclusion criteria. If inclusion criteria were met, staff collected address information for mailing the in-home questionnaire and scheduled an appointment for an in-home interview. In step three of the study, in-home interviews were completed with caregivers and elders where eligible, using a computer assisted live interview technique.

Inclusion criteria for the study required that: (1) elders be 60 years of age or older, (2) have a diagnosis of AD according to the NINCDS/ADRDA criteria or dementia of Alzheimer's type according to the DSM-III-R or DSM-IV in the 3 years prior to their enrollment in the study, (3) possess the ability to speak English, and (4) achieve a score of 16 or greater on the Folstein Mini Mental Status Exam (MMSE.) Inclusion criteria for caregivers required that: (1) caregivers be a family member, (2) with the ability to speak English, (3) provide 20 hours a week or more of care, and possess a phone. 

Persons who did not meet the inclusion criteria were excluded from participating in the study with the exception of elders who met all criteria except requisite MMSE score. In these cases a surrogate was used to provide answers on an elder's behalf providing that the surrogate felt close to the elder, had met at least one time per week with the elder over the past year, and was not the primary caregiver.

Requisite MMSE score was lowered from the standard of 18 to 16 or higher, and data collection was limited to persons diagnosed within the 3-year period prior to enrollment to reduce the number of patients who were likely to have surpassed the cognitive decline cut-off. This was also an effort to decrease the response bias that a large number of surrogates might induce. Of the 76 elders who participated, only 17 required the inclusion of surrogate information.

### 2.2. Tests and Measures

The selection of measures used to determine the characteristics that place a family at risk for, or are protective against physical violence were based on formal criteria. The selection of measures had to: (1) reflect proposed theoretical risk factors for the increased risk or prevention of violence and aggression as based on previous work on elder abuse in the general population, or specific work on violence and aggression in family members with AD; (2) represent a conceptual and logical indicator of the construct being assessed; (3) exhibit strong to moderate reliability and validity as evidenced by formal testing; (4) be relatively short and easy to administer with an economy of time; and (5) have a history of use with diverse samples. 

The Conflict Tactic Scale (CTS) was the principal measure of elder mistreatment in the AV_CAD, and the violence subscale served as the principal outcome measure of this study. The CTS was originally developed by Straus and his colleagues for use in their seminal study on violence in the American family [74]. This instrument was chosen because of its frequency of use in the study of violence in families [9], as well as by studies that have looked at violence in AD families on a smaller scale [23]. It includes items in compliance with the definition for physical abuse such as “pushed, shoved, or grabbed” and “slapped, and spanked”, as measured through caregivers' reports of their own behavior and caregivers' reports of elders behavior, and it offers a dichotomous way to measure the presence or absence of physical mistreatment.

Independent variables included measures of risk and vulnerability as outlined in Figure 1. Measures of vulnerability on the part of the caregiver included elder's demographic information such as age, gender and race; elder's cognitive status as measured by the number of dementia symptoms present; elder's functional status as measured by the “Elders Level of Impairment” subscale of the DON-R; psychological status as measured by the Langer Psychiatric Symptoms Scale [75] and the Cornell Depression Scale, CDS [76]; and the elders health status by the number of drugs prescribed for the treatment of health conditions. Measures of risk included caregiver's demographic information such as age, gender, and race; caregiver's cognitive status as measured by the Folstein Mini Mental Status exam, MMSE [77]; caregiver burden as measured by the “Patients unmet need for care” subscale of the Determination of Need Scale Revised, DON-R [78], the Caregiver Hassle Scale, CHS [79], and subjective report of caregiver burden; and caregiver functional status as measured by the level of impairment subscale of the DON-R. Additional measures of caregiver risk included the presence of social support as measured by the Norbeck Social Support Scale, NSSS [80]; caregiver psychological health as measured by the Langer Psychiatric Symptoms Scale [75], the Center for Epidemiological Studies depression scale, CES-D [81], the Rosenberg Self Esteem Scale, RSE [82], and the Michigan Alcohol Screening Test, MAST [83]; and caregiver coping as measured by the Coping Styles Questionnaire, CSQ [84].

### 2.3. Data Analysis

Data were collected through mailed questionnaire and in-home interview. Data collected during the in-home interview were entered immediately into an Epi-Info database format via laptop computer, and later transformed into an SPSS useable format using Stat Transfer 6.0 software. Data collected via mailed questionnaire were entered directly into an SPSS 11.0 database for data analysis.

For the purpose of this analysis, demographics of the overall sample were computed, and a step-wise logistic regression model was used to test for variables significantly associated with physical mistreatment of elders. All independent variables were entered into the model on step one. Variables were considered to be significant protective or risk factors if they were found significant on step one, and if they remained significant when tested against all other significant variables in the model on step two. All variables were tested for multicollinearity prior to the running of the regression models.

## 3. Results

In all, 254 caregivers (see Table 1) and 76 elders (See Table 2) participated in the study. Caregivers had a mean age of 63.84 years (±13.07 years) and were primarily wives (34.2%) and children (33.3%), followed by husbands (18.5%). Care-recipients had a mean age of 78.57 years (±8.41 years) and 59% were female. Both care-recipients and caregivers were 85% Caucasian, 10.3% Hispanic, and approximately 4.5% African American. Forty-four percent of caregivers were non-Roman Catholic Christians, as were 43.2% of elders. An additional 26.1% of caregivers and elders were Roman Catholics. Of those, 21.7% had household incomes between $20,000 and $29,000. For care-recipients who do not live with their caregivers, many are living in poverty. Thirty-five percent have incomes less than $20,000 a year. Forty-nine percent of caregivers were providing care to someone with 11–15 dementia symptoms, 84.3% reported a subjective feeling that providing care to the elder was a burden, and 41.9% were seriously considering the need for nursing home placement in the future. Only 17.4% of caregivers and 25.9% of patients reported no level of depression. Twenty-six percent of caregivers were mildly depressed, 37% were moderately depressed, and 20% reported severe depression as measured by the self-report CES-D. Interestingly, only 7% of caregivers reported low self-esteem as measured by the Rosenberg self esteem scale. Minor depression was reported in 29.6% of elders, possible major depression in 16.2% of elders, probable major depression in 19.9% of elders, and definite major depression was reported in 8.3% of elders as measured by the Cornell Depression Scale, which identifies depression in elders based on caregivers responses.

The use of violence was self-reported by 17.2% of caregivers, and was reported as a technique used against them by 26.1% of elders. Upon comparison of caregivers who used violence as a conflict resolution strategy with those who did not (see Table 3), logistic regression analyses indicated that caregivers who were providing care to elders with high levels of functional impairment (*P* = .034; OR = 2.049; CI = 1.093–4.912) were twice as likely to be violent with their care recipient than those who were not. Caregivers with alcohol problems were 3 times as likely to be violent with elders for whom they were providing care (*P* = .041; OR = 3.217; CI = 2.382–4.775), and those providing care to elders with greater numbers of dementia symptoms (*P* = .019; OR = 4.817; CI = 3.509–12.518), or to elders who used violence (*P* = .010; OR = 4.168; CI = 2.176–8.399) were four times as likely to be violent with the elder in their care. Caregivers with high self-esteem were significantly less likely to engage in violent behavior (*P* = .046; OR = .662; CI = .591–.748). Additionally, the interaction effect of risk and vulnerability was found to result in greater likelihood for physical mistreatment. The interactions between dementia symptoms displayed on the part of elders and depression in caregivers (*P* = .007; OR = 5.483; CI = 3.217–7.075), and dementia symptoms and violent behavior on the part of elders, and alcoholism on the part of caregivers (*P* = .052; OR = 6.176; CI = 4.511–9.706), were significantly related to the likelihood of physical mistreatment. Depressed caregivers providing care to elders with more dementia symptoms were five times as likely to engage in violent behavior as those who were not. Caregivers who abused alcohol, and who were providing care to violent elders with high levels of dementia symptoms were six times as likely to engage in violent behavior as those who did not. The model accounted for 48.3% of the variance (Nagelkerke *R* square = .483). The Homer-Lemeshow goodness of fit test was not significant (*χ* ^2^ = 9.628; df = 8; *P* = .365) and indicates that the model is a good fit.

## 4. Discussion

This paper has revealed several factors such as self-esteem and alcohol abuse in caregivers, and violence, dementia symptoms, and functional impairment in elders, that increase the likelihood of physical mistreatment. That cognitive impairment is a factor in elder mistreatment has been suggested by other researchers [23, 26, 36, 41, 43–45, 85] and is clearly supported by this research in light of the increased numbers of persons in this group who report physical violence (43 in 254) compared to persons in the population at large (20 in 1000) [9]. While persons may be protected from mistreatment at the onset of their disease when they show few symptoms, AD is a progressive dementing disorder, which gradually results in more cognitive difficulties. As a result, it is likely that as their disease progresses their risk goes up. In addition as a direct result of the etiology of the disease, as it progresses, persons with AD become more functionally impaired, may fail to recognize loved ones, lose their social filters, and become more verbally and physically combative. As a consequence, they may become more physically abusive and receive more physical abuse. This pattern exemplifies cases of reciprocal violence and may explain the largest of what is likely two subgroups of persons who are mistreating: those who are engaging in violent behavior as a direct result of having violence used against them, and those who are engaging in violent behavior unprovoked. While this data set did not contain sufficient numbers of persons engaging in non-reciprocal violence to examine this population with scientific certainty (*N* = 17), future research should separate cases of caregiver violence in which elders are physically violent and those cases in which elders are not, as it is likely that they represent two differing risk profiles. With a larger sample gathered from several geographic regions it is likely that sufficient cases could be gathered to examine this question.

Psychological health of the perpetrator was also found to be a significant predictor of physical violence in caregivers. Those with who abused alcohol were three times more likely to engage in physical violence than those who did not. This research supports other research findings that substance abuse increases risk of elder mistreatment [31, 33, 36, 38] and suggests that screening for this condition by health professionals may contribute to an avenue of prevention. Additionally, since higher levels of functional impairment resulted in twice the risk for physical mistreatment, avenues for prevention may lie in increased physical, occupational, and assistive device therapies for older adults, as studies have shown that many older adults are significantly below their functional capacity despite the presence of physical impairment [67].

That many of these factors contribute to physical mistreatment in older adults is not altogether surprising. It makes intuitive sense that those least able to defend themselves as a result of functional impairment, or those least able to report as a result of factors such as cognitive impairment will have the greatest likelihood of mistreatment. The unique aspect of this research is the theoretically supported decision to look at risks and vulnerabilities in older adults with AD and their caregivers simultaneously, and to discover in doing so that the interaction of elders “vulnerabilities” and caregiver “risk” results in an even greater likelihood abuse will happen. Elders with high levels of dementia symptoms being cared for by caregivers with low self-esteem were five times more likely to be victims of physical mistreatment than those who were not. Additionally, violent elders with dementia being cared for by a caregiver with alcohol problems were six times more likely to be physically mistreated compared with the fourfold risk of dementia symptoms on its own. While determining whether or not alcoholism stems from caring for someone who is violent, or whether alcoholism in caregivers contributes to violence in elders is beyond the scope of this paper, what this research demonstrates is the need for models that account for both victim and perpetrator characteristics, as well as the interaction between them such as that posited by the Risk and Vulnerability model used here.

Future research should include other risk factors such as familial history, behavioral acculturation, and state-trait anger which may also contribute to physical mistreatment and should include, if possible, a random sample of older adults over a larger, more diverse, geographic region. This would generate larger samples which would allow for the testing of cases of physical mistreatment versus those who do not verbally or physically mistreat, as well as for the testing of differences within mistreatment subgroups (i.e., those who involve instances of reciprocal violence and those that do not). Additionally, while the consequences of elder mistreatment have been found to include emotional difficulties such as depression [56, 64, 65], feelings of inadequacy and self-contempt [37, 59, 60], impaired life functioning [55, 56], and health problems such as immunological dysfunction [56, 57, 63, 65, 69, 70], and increased mortality [66–68], no study has examined the long term consequences of physical mistreatment in persons with Alzheimer's disease specifically. While elder mistreatment may result in a threefold increase in mortality in persons over the age of 65 as a whole [66, 68] it may result in even greater effects for persons with AD.

## 5. Conclusion

Caring for a loved one with Alzheimer's disease is a difficult job. Alzheimer's disease is a progressive dementing disorder that lasts from 5 to 15 years. It starts with difficulties in cognition and ends with a complete loss of ability to complete most activities of daily living unassisted. As health care professionals, we have a duty to monitor the quality of life in these persons to prevent abuse where possible. The closer we come to a risk profile, the easier this task will be. This study also suggests that continued funding for research at this level of detail, as suggested by the National Research Council report on elder abuse [48], is imperative if useful, specific interventions are to be implemented.

## Figures and Tables

**Box 1:**
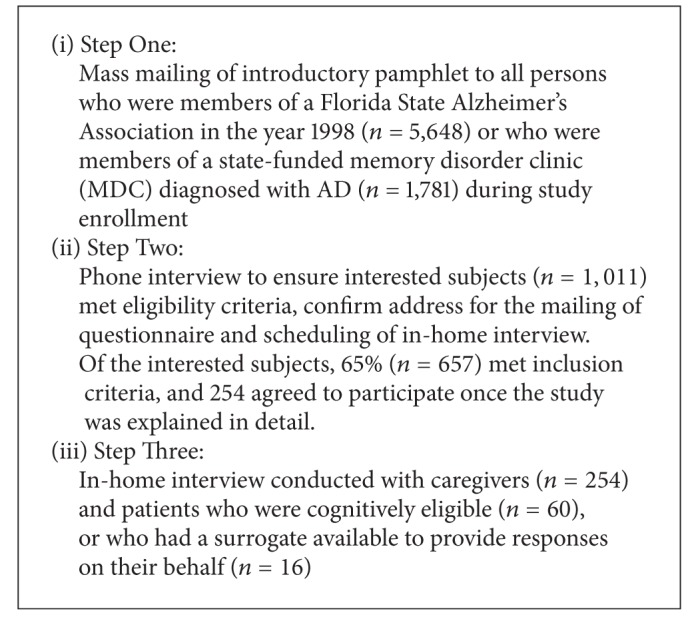
Data collection.

**Table 1:** Caregiver demographics (*N* = 254).

Mean age	63.84 (±13.07)
Age by group	
21–30	0.5%
31–40	5%
41–50	9.9%
51–60	27%
61–70	19.8%
71–80	28.8%
81–90	8.6%
90+	0.5%
Gender	
Female	74.8%
Male	25.2%
Ethnicity	
Caucasian	85.1%
African American	4.6%
Hispanic	10.3%
Religion	
Roman Catholic	26.1%
Other Christian	44.1%
Jewish	15.8%
Other	14.0%
Relationship to patient	
Wife	34.2%
Husband	18.5%
Child	33.2%
Other	14.0%
Income^1^	
Under $19,999	10.9%
$20,000–$29,999	18.6%
$30,000–$39,999	13.2%
$40,000–$49,999	12.3%
$50,000+	45.0%
Subjective feeling of burden	
Yes	84.3%
No	15.7%
Psychiatric symptoms	
0-1	24.9%
2-3	19.2%
4-5	23.2%
6+	32.5%
Level of depression	
Non-depressed	17.4%
Mildly depressed	25.7%
Moderately depressed	36.7%
Severely depressed	20.2%
Level of Self Esteem	
Moderately low	0.9%
Low	6.0%
High	18.1%
Moderately high	30.2%
Very high	44.7%
Considering nursing home	
Not really considering	31.4%
Considering	26.7%
Seriously considering	41.9%
Physically violent^2^	
Yes	17.2%
No	82.8%

^1^Figures represent combined household income.

^2^As measured by the Violence sub-scale of the CTS.

**Table 2:** Elder demographics (*N* = 76).

Mean age	78.57 (±8.41)
Age by group	
60–70	14.5%
71–80	43.4%
81–90	36.7%
90+	5.4%
Gender	
Female	59%
Male	41%
Ethnicity	
Caucasian	84.8%
African American	4.5%
Hispanic	9.4%
Other	1.3%
Religion	
Roman Catholic	26.1%
Other Christian	43.2%
Jewish	15.8%
Other	14.9%
Income^1^	
Under $19,999	35.5%
$20,000–$29,999	15.7%
$30,000–$39,999	11.4%
$40,000–49,999	8.5%
$50,000+	28.9%
Income^2^	
Under $19,999	15.2%
$20,000–29,999	21.7%
$30,000–$39,999	11.6%
$40,000–49,999	13.8%
$50,000+	37.6%
Dementia Symptoms	
1–5 symptoms noticed	1.3%
6–10 symptoms noticed	24.1%
11–15 symptoms noticed	49.1%
16–20 symptoms noticed	25.4%
Depression	
No depression	25.9%
Minor depression	29.6%
Possible major depression	16.2%
Probable major depression	19.9%
Definite major depression	8.3%
Number of drugs taken	
None reported	79.2%
1-2	5.0%
3-4	5.4%
5-6	3.5%
Verbally Aggressive^3^	
No	25.2%
Yes	74.2%
Violent^4^	
Yes	73.9%
No	26.1%

^1^Represents household income when not living with caregiver.

^2^Represents household income when living with caregiver.

^3^As measured by the Verbal Aggression sub-scale of the CTS.

^4^As measured by the Violence sub-scale of the CTS.

**Table 3:** Logistic regression analysis: violent caregivers versus all others (*n* = 254).

Variable	*B*	S.E.	Sig.	EXP(*B*)	95% C.I. for EXP(*B*)	
					Lower	Upper
Patient vulnerability						
Age	.564	.555	.309	1.758	.593	5.214
Gender	−.202	.972	.048	.817	.422	.949
Race	9.569	27.669	.999	14.274	.437	8.453
Number of dementia symptoms	2.470	1.050	.019	4.817	3.509	12.518
Level of functional impairment	1.231	.581	.034	2.049	1.093	4.912
Depression	−.634	.423	.134	.530	.231	1.216
Number of drugs taken	.463	.538	.390	1.589	.553	4.563
Verbal aggression	2.129	1.450	.142	.119	.007	2.841
Violence	3.185	1.228	.010	4.168	2.176	8.399
Caregiver risk						
Age	.355	.382	.352	1.426	.675	3.013
Sex	−1.497	1.471	.309	.224	.013	3.998
Race	−8.897	27.669	.999	1.010	.653	8.976
Hassle experienced by caregiving	.173	.234	.460	1.189	.751	1.880
Level of impairment sub-scale	−14.523	54.843	.996	4.962	.655	9.877
Cognitive status	.953	.800	.233	2.594	.541	12.435
Level of social support	.023	.418	.956	1.023	.451	2.322
Psychiatric symptoms	.249	.486	.608	1.283	.495	3.326
Depression	.872	.535	.103	2.391	.837	6.825
Self esteem	−.472	.514	.046	.662	.591	.748
Alcoholism	3.562	2.308	.041	3.217	2.382	4.775
Coping style	−17.491	33.673	.999	2.534	.544	5.436
Interaction of risk and vulnerability						
Pt dementia ∗ cg depression	1.728	.408	.007	5.483	3.217	7.075
Pt depression ∗ cg hassle ∗ Cg psychiatric symptoms	.162	.425	.052	6.176	4.511	9.706
Constant	−38.572	26.066	.139	.000		

(a) Step wise logistic regression model: variables were considered significant risk factors if they were found significant on step one and if they remained significant when tested against all other significant variables in the model onstep two. All variables found significant on step 1 remained significant at step 2.

(b) Model accounts for 48.3% of the variance. Nagelkerke *R* square = .483.

(c) Homer-Lemeshow goodness of fit test (*X* ^2^ = 9.628, df = 8, *P* = .365).
